# Serum Cytokine Profiles in Patients with Dengue Fever at the Acute Infection Phase

**DOI:** 10.1155/2018/8403937

**Published:** 2018-01-30

**Authors:** Junyuan Huang, Weiwen Liang, Shaoyan Chen, Ye Zhu, Haiming Chen, Chris Ka Pun Mok, Yingchun Zhou

**Affiliations:** ^1^First Affiliated Hospital of Guangzhou University of Chinese Medicine, Guangzhou, Guangdong, China; ^2^State Key Laboratory of Respiratory Disease, National Clinical Research Center for Respiratory Disease, First Affiliated Hospital of Guangzhou Medical University, Guangzhou, Guangdong, China; ^3^HKU-Pasteur Research Pole, School of Public Health, HKU Li Ka Shing Faculty of Medicine, The University of Hong Kong, Hong Kong

## Abstract

**Background:**

Dengue virus (DENV) is transmitted by mosquito and has been circulating in Guangdong, China, for over 30 years. Dengue infection causes mild to severe disease symptoms in human. Cytokine profiles were suggested to be crucial especially during the acute stage in the dengue infection.

**Aim:**

To determine the cytokine profiles at the acute stage in patients with primary or secondary dengue infection in Guangzhou city in the 2014 outbreak.

**Methods:**

We investigated 23 inflammatory cytokines in serum collected from dengue-infected patients and analyzed their correlations with their clinical indexes.

**Results:**

The concentrations of CXCL9, IP-10, CXCL11, IL-8, IL-10, and CCL2 in serum were significantly higher in the groups of DENV-infected patients during the first two weeks than those of control group while CCL17 and CXCL5 showed lower expression level in the patients. Among these cytokines, CXCL9, CCL17, and CXCL5 showed statistical difference between the groups of primary and secondary infections. The platelet count and lactate dehydrogenase were correlated with the level of CCL17 and MIP-1*α*/CXCL5, respectively, in the group of secondary infection.

**Conclusions:**

We determined the cytokine profiles in serum of the patients during the 2014 dengue outbreak. The expression of specific cytokines was associated with the secondary infection.

## 1. Introduction

Dengue virus (DENV) as a member of *Flaviviridae* family is an enveloped, positive-sense, single-strain RNA virus with the size of around 11 kb. It is mainly transmitted by *Aedes albopictus* resulting in dengue fever (DF) and, in some cases, progresses to dengue hemorrhagic fever (DHF) and dengue shock syndrome (DSS). The dengue outbreaks frequently occurred in tropical and subtropical climates areas. A report indicated that there were around 96 million with apparent dengue infections and 294 million with asymptomatic infections globally in 2010 [1]. During 2010 to 2015, around 15.7% of DENV-infected cases developed DHF and 0.7% of DENV-infected cases died globally. In endemic areas, many people have been infected by dengue virus more than once in their entire life. Indeed, around 55.9% of DENV-infected cases were known as secondary infection [2]. The nonneutralizing antibodies against heterotypic DENV in secondary infection was thought to be partially responsible for the severe dengue due to the antibody-dependent enhancement (ADE) [3]. However, many cases of primary infection could also progress to severe dengue fever.

Cytokines are a group of protein which serve as signaling transducer and modulator to regulate the host immunity especially during infection. Numbers of studies have investigated the cytokine profiles in the serum samples of the dengue-infected patients in order to understand the immune responses against this pathogen. Importantly, some studies have figured that induction of several cytokines was associated with severe dengue infection [4–7].

In mainland China, outbreaks of dengue fever mainly occurred in the southern area like Guangdong or Yunnan [8, 9]. Epidemiological studies showed that dengue 1 was the major serotype circulating in Guangdong province although small percentage of patients were infected by other three serotypes over the last 20 years which were suggested as imported incidents [10]. The most recent outbreak in Guangdong province occurred in 2014 in which around fifty thousand of dengue fever cases were reported and six patients died [11]. While majority of the patients were infected by dengue 1 viruses in this outbreak, we and others previously showed that the condition of the patients were generally mild compared to the previous large-scale outbreaks [12]. However, little was known about the cytokine profiles in these patients during the acute stage of the dengue infection. In this study, we investigated the levels of 23 inflammatory cytokines in serum from patients with either primary or secondary dengue infection during the acute phase in the 2014 dengue outbreak. In addition, their correlations with the routine clinical indexes were also analyzed.

## 2. Materials and Methods

### 2.1. Study Population and Sample Collection

During the dengue epidemics period from August to December 2014, hospitalized patients who were laboratory confirmed by either (i) DENV RNA real-time PCR (DAAN, China), (ii) DENV antigen NS1 ELISA (KeShun, China), or (iii) DENV IgM ELISA (Panbio, Australia) at the First Affiliated Hospital of Guangzhou University of Chinese Medicine were enrolled. Patients were categorized as primary infections or secondary infections according to the IgM/IgG ratio and the patients were defined as DF or severe DF according to the guideline by WHO in 2009 [13]. A total of 297 DENV-infected patients and 20 healthy controls were included in this study. Those with any disease symptom (such as fever, cough, obstruction, or other unwellness) that indicated the infection or those with any immunocompromised disease were excluded from the control group. Serum and peripheral blood samples were obtained from patients at different day after the onset day of dengue fever (day 1). Samples were further grouped to those collected in the first week (day 1 to day 7) or second week (day 8 to day 14). The patient identities were delinked from the samples during analysis. Approval for the study was obtained from the ethics committee of the First Affiliated Hospital of Guangzhou University of Chinese Medicine, and informed consent was waived by the committee.

### 2.2. Detection of Clinical Routine Indexes

Clinical routine indexes including count of white blood cells (WBC), platelet (PLT), and hematocrit (HCT) were determined from the peripheral blood. Glutamic-pyruvic transaminase (ALT), aspartate transaminase (AST), lactate dehydrogenase (LDH), creatine kinase (CK), creatinine (Cr), and urea were detected from the serum samples which were isolated from clotted blood by centrifugation.

### 2.3. Quantification of Inflammatory Cytokines

Inflammatory cytokines were identified and quantified using the flow cytometry cytokine bead array purchased from LEGENDplex (BioLegend, CA, USA) according to the manufacturer's instruction. Twenty-three cytokines were included: IFN-*α*, IFN-*γ*, TNF-*α*, IL-6, IL-8, IL-10, IL-1*β*, IL-18, IL-23, IL-33, IL-17A, IL-12, CXCL9, CXCL11, CXCL5, CCL2, CCL11, CCL17, CCL20, MIP-1*α*, MIP-1*β*, IP-10, and RANTES. Briefly, the cytokines were captured by beads which were conjugated with specific biotinylated antibodies. The streptavidin-phycoerythrin was subsequently added and bonded to the biotinylated antibodies, providing fluorescent signal intensities in proportion to the amount of bound analytes. The cytokines were identified by the size of beads and PE fluorescent signal and quantified according to a standard curve on BD FACSVerse flow cytometer (BD, USA).

## 3. Statistical Analysis

Statistical analysis was performed using SPSS 23.0 and GraphPad Prism v7.0. The mean values of clinical routine indexes and cytokines in each cohort were compared using Mann–Whitney *U* test or *t*-test. The correlations between routine indexes and cytokines were analyzed based on the Spearman rank correlation coefficient (*R* value). A *P* value <0.05 was considered as statistically significant.

## 4. Results

### 4.1. General Background and Clinical Routine Indexes

In 297 laboratory-confirmed DENV-infected patients, 234 patients were classified as primary infection while 63 patients were classified as secondary infection, without any cases developed severe dengue fever. The mean age of the patients with secondary infection was higher than those with primary infection (58.37 ± 18.874 versus 45.99 ± 18.369, *P* < 0.001) as presented in Table 1. The laboratory diagnosis results showed that the levels of WBC, PLT, HCT, and UREA were lower in all DENV-infected patients while ALT, AST, LDH, and CK were higher compared to the control group. However, no statistical difference was observed in the clinical routine indexes between the groups of primary infection and secondary infection (Table 2).

### 4.2. Inflammatory Cytokines in DENV Primary Infections and Secondary Infections

**Figure 1:**
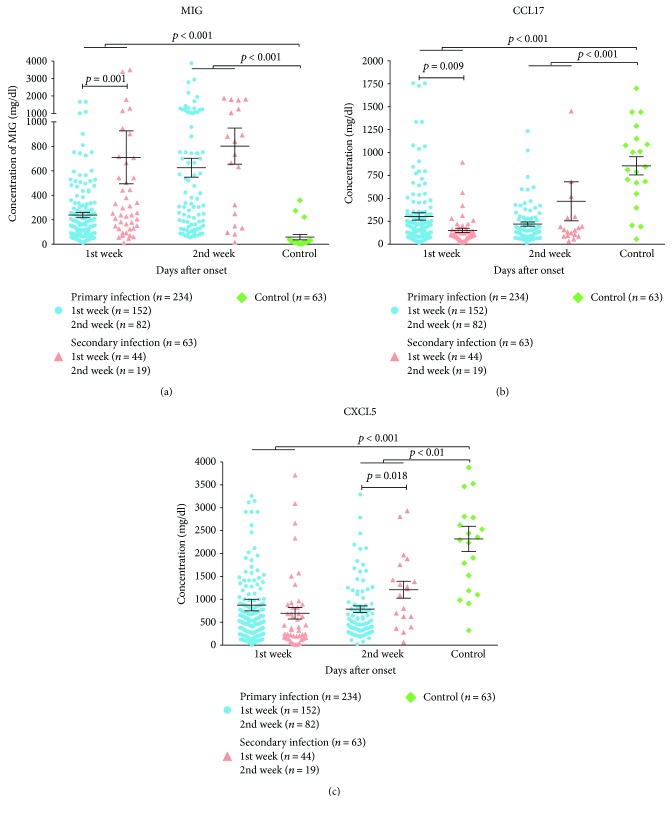
Inflammatory cytokines with statistical differences between primary infections and secondary infections were showed. *P* values obtained from Mann–Whitney *U* test were annotated.

Among 23 inflammatory cytokines detected, the concentrations of IL-6, IL-1*β*, IL-33, IL-23, IL-17A, IL-12, IFN-*α*, and TNF-*α* were lower than the detection limit that we could not conclude their expression. The remaining 15 inflammatory cytokines were further quantified and shown in Table 3. The concentrations of CXCL9, IP-10, CXCL11, IL-8, IL-10, and CCL2 in the serum were significantly higher in the groups of DENV-infected patients during the first two weeks than the control group (*P* < 0.05). On the contrary, the levels of CCL17 and CXCL5 were lower in the DENV-infected patients compared to the controls. Among these cytokines, we found that CXCL9, CCL17, and CXCL5 showed statistical difference between the groups of primary and secondary infections (Figure 1). During the 1st week, the concentration of CXCL9 was higher in secondary infections than that in primary infections (710.20 ± 1435.59 versus 238.61 ± 265.29), while CCL17 was lower in secondary infections than that in primary infections (151.09 ± 155.53 versus 303.26 ± 490.43). During the 2nd week, the concentration of CXCL5 was higher in secondary infections than those in primary infections (CXCL5: 1209.47 ± 804.15 versus 786.72 ± 649.12).

### 4.3. Correlation between Clinical Routine Indexes and Inflammatory Cytokines

We next tested the correlation between the routine clinical indexes and inflammatory cytokines in DENV infection. We found that several cytokines showed statistically significant correlation with clinical routine indexes (Table 4). In the group of all dengue-infected patients or primary infection, there were only weak correlation in specific cytokines with urea, AST, or LDH. However, PLT was found to be strongly negative correlated with the level of CCL17 (*R* = −0.31, *P* = 0.012) in the group of secondary infection. In addition, the level of LDH was also significantly associated with the expression of MIP-1*α* (*R* = 0.49, *P* = 0.001) and CXCL5 (*R* = 0.39, *P* = 0.015).

## 5. Discussion

Proinflammatory cytokines were secreted to initiate the inflammation and to control the DENV replication especially at the early stage of infection. However, dysregulation of these cytokines was also considered as important reason in dengue pathogenesis especially in DHF and DSS [14, 15]. In this study, we have focused to investigate the cytokine induction profiles from the patients infected with dengue viruses during the Guangdong outbreak in 2014. We also compared their levels between the patients with primary and secondary infections as well as the clinical implications. Interestingly, we found that while several proinflammatory cytokines such as CXCL9, IP-10, CXCL11, IL-8, IL-10, and so on were highly upregulated in the patients after dengue infection, the levels of CCL17 and CXCL5 were significantly lower than the controls. These results provided information for us to understand the interplay between the virus and the host responses during the acute stage of dengue infection. CXCL9, IP-10 (CXCL10), and CXCL11 are the ligands of CXCR3. They are chemoattractants of T lymphocytes and NK cells and make contribution in terms of antiviral and antimicrobial [16–18]. Specifically, CXCR3-ligand interaction attracts Th1 cells and promotes Th1 cell maturation. Recent study demonstrated that there was an increase of T cell activation in asymptomatic viremia compared to those with clinical dengue [19]. In addition, CXCL10 (IP-10) was shown to compete with dengue virus for binding to cell surface heparan sulfate and led to a reduction of dengue infection to cells [20–22]. Our results indicated that the CXCR3-related pathways were crucial at the acute phase DENV infection, and T cell responses may be one of the key immune component to regulate the dengue infection. However, dysregulation of these CXCR ligands was suggested to associate with the disease severity. For example, CXCL9 was identified as a risk factor of increasing vascular permeability in DENV infection [14]. It has been also demonstrated that IP-10 was more elevated in patients who developed dengue hemorrhagic fever or dengue shock syndrome compared to the less severe controls [23]. Although the IP-10 and CXCL9 are considered associated with increase in vascular permeability which leads to severe dengue fever, they are only consider as the risk factors contributed to the severity of the dengue infection. The entire immune environment which contained additional factors in regulation of other cytokines may also play a crucial role in progression of diseases. Indeed, we do not know if the concentrations of the two cytokines were high enough in the patients to lead to the severe outcome as we did not include the patients with severe dengue fever in our study. On the contrary, CCL17 is constitutively expressed in thymus and plays similar role as CXCL9 in inflammation. However, the concentration of CCL17 in primary and secondary infections of this study were lower than that in controls. Wong et al. figured that CCL17 deficiency in mice could be a protective factor to prevent severe colitis by reducing production of other proinflammatory cytokines [21]. Moreover, CCR4, as the only known receptor for CCL17, was also found to markedly decreased the tissue injury in DENV infected mice when it was blocked [24]. These results suggested that reduction of CCL17 may benefit to the host during dengue infection. The functions of CXCL5 have been well studied in cancer and bacterial infection but rarely and ambiguous in dengue. The downregulated CXCL5 may play similar role with CCL17 in DENV infection.

Previous studies have found a significant increase in IFN-*γ* and TNF-*α* in the patients with dengue infection [4, 7]. Both of them are important proinflammatory cytokines and have been shown to associate with the disease severity. While the upregulation of the IFN-*γ* can induce plasma leakage, TNF-*α* may indicate for the activation of vascular endothelial cell and may increase vascular permeability which lead to hemorrhagic fever [25–27]. In our study, we did not observe a detectable level of TNF-*α* from the serum of our patients collected from the first two weeks of infection. In addition, highly varied level of IFN-*γ* in the serum was also found at the first week. It is likely that the mild condition of the patients does not necessary trigger these immune responses given the fact that only very small proportion of patients developed severe forms of dengue fever in this outbreak. In addition, it has been noted that the levels of IFN-*γ* and TNF-*α* varied among different studies. Comparable levels of these two cytokines between patients and healthy controls have also been reported in some studies [28, 29]. It has been known that CXCL9, IP-10 (CXCL10), and CXCL11 are all IFN-*γ*-induced chemokines. The observation of lacking IFN-*γ* induction could not explain why there were still high level of CXCR3 ligands expressed in the serum. These CXCR3 ligands may be regulated by other stimuli such as IFN-*β* which was not included in this study.

One of the limitation in our study was that we did not know the serotype of the dengue virus in the patients. Also, we could not identify the previous infection history of those patients with secondary infection. Thus, the impact of the serotype on the cytokine profiles was not clear. However, epidemiological data suggested that serotype 1 was the major subtype circulated in Guangdong province over the last 20 years and large proportion of patients were also infected by dengue 1 in the 2014 outbreak [10]. It is thus logical to postulate that the results we obtained should mainly reflect the impact of dengue 1 infection. Furthermore, we could not compare the cytokine profile between mild and severe dengue patients as there were no severe patients admitted to our hospital. Despite of these limitations and there are some similar studies that reveal the cytokine profile in dengue-infected patients, our study has done a more comprehensive detection of 23 cytokines in up to 297 infected patients. Our results not only extended our understanding on the immunological features of the patients in the Guangzhou dengue outbreak in 2014 but also determined the association of clinical routine indexes and these inflammatory cytokines which provide new insight to the pathogenesis of dengue fever in human.

## Figures and Tables

**Table 1:** General background.

	Primary infections (A)	Secondary infections (B)	Controls	*P* value
				A versus B
Age (mean ± SD)	45.99 ± 18.369	58.37 ± 18.874	38.25 ± 7.468	<0.001^∗∗^
Gender				
Male	112	31	9	0.888
Female	122	32	11	

^∗∗^ *P* < 0.05.

**Table 2:** Clinical routine indexes in infections and controls.

	Primary infections (mean ± SD)		*P* value		Secondary infections (mean ± SD)		*P* value		Controls (E) (mean ± SD)	*P* value	
	1st week (A)	2nd week (B)	A versus E	B versus E	1st week (C)	2nd week (D)	C versus E	D versus E		A versus C	B versus D
Number of case	151	82	—	—	44	19	—	—	20	—	—
WBC (×10^9^/L)	4.02 ± 2.57	3.79 ± 2.58	<0.001^∗∗^	<0.001^∗∗^	3.80 ± 1.83	4.00 ± 1.65	<0.001^∗∗^	<0.001^∗∗^	7.30 ± 1.51	0.840	0.242
PLT (×10^9^/L)	104.07 ± 61.90	100.46 ± 59.68	<0.001^∗∗^	<0.001^∗∗^	105.23 ± 65.66	112.79 ± 74.43	<0.001^∗∗^	<0.001^∗∗^	248.70 ± 63.40	0.983	0.599
HCT	0.39 ± 0.05	0.39 ± 0.04	<0.001^∗∗^	<0.001^∗∗^	0.40 ± 0.04	0.39 ± 0.05	<0.001^∗∗^	<0.001^∗∗^	0.44 ± 0.04	0.366	0.562
ALT (U/L)	62.54 ± 79.63	62.72 ± 63.17	<0.001^∗∗^	<0.001^∗∗^	54.57 ± 71.40	74.22 ± 73.40	<0.001^∗∗^	<0.001^∗∗^	19.70 ± 8.25	0.679	0.316
AST (U/L)	76.60 ± 64.87	76.48 ± 63.68	<0.001^∗∗^	<0.001^∗∗^	77.16 ± 57.99	109.78 ± 148.19	<0.001^∗∗^	<0.001^∗∗^	18.50 ± 3.59	0.799	0.618
LDH (U/L)	297.16 ± 154.33	309.37 ± 111.52	<0.001^∗∗^	<0.001^∗∗^	322.97 ± 170.13	535.00 ± 502.20	<0.001^∗∗^	<0.001^∗∗^	151.40 ± 15.41	0.303	0.467
CK (U/L)	235.27 ± 262.68	273.98 ± 378.99	<0.001^∗∗^	<0.001^∗∗^	370.50 ± 500.50	3651.00 ± 10569.53	<0.001^∗∗^	0.009^∗∗^	74.55 ± 18.19	0.233	0.867
Cr (*μ*mol/L)	84.38 ± 50.42	86.96 ± 60.76	0.056	0.068	82.68 ± 26.19	78.53 ± 11.85	0.072	0.094	71.45 ± 12.95	0.897	0.868
UREA (mmol/L)	3.71 ± 2.20	3.84 ± 1.92	0.001^∗∗^	0.003^∗∗^	3.48 ± 1.83	3.25 ± 1.38	0.001^∗∗^	0.002^∗∗^	4.64 ± 1.18	0.318	0.178

Mean ± SD of clinical routine indexes were presented and performed analysis using Mann–Whitney *U* test or *t*-test between two cohorts. The statistical significance is indicated by asterisks: ^∗∗^*P* < 0.05.

**Table 3:** Concentration of inflammatory cytokines in infections and controls.

	Primary infection (mean ± SD)		*P* value		Secondary infection (mean ± SD)		*P* value		Control (E) (mean ± SD)	*P* value	
	1st week (A)	2nd week (B)	A versus E	B versus E	1st week (C)	2nd week (D)	C versus E	D versus E		A versus C	B versus D
Number of case	152	82	—	—	44	19			20		
CXCL9 (MIG)	238.61 ± 265.29	625.52 ± 700.20	<0.001^∗∗^	<0.001^∗∗^	710.20 ± 1435.59	802.32 ± 644.12	<0.001^∗∗^	<0.001^∗∗^	58.35 ± 100.68	0.001^∗∗^	0.225
CCL17 (TARC)	303.26 ± 490.43	220.40 ± 209.15	<0.001^∗∗^	<0.001^∗∗^	151.09 ± 155.53	468.16 ± 926.50	<0.001^∗∗^	0.001^∗∗^	855.40 ± 442.74	0.009^∗∗^	0.537
CXCL5 (ENA-78)	875.13 ± 1553.74	786.72 ± 649.12	<0.001^∗∗^	<0.001^∗∗^	695.59 ± 833.55	1209.47 ± 804.15	<0.001^∗∗^	0.002^∗∗^	2320.05 ± 1237.33	0.229	0.018^∗^
CCL11 (eotaxin)	341.20 ± 209.82	362.48 ± 233.38	0.385	0.604	307.23 ± 142.02	468.58 ± 163.38	0.389	0.032^∗^	375.65 ± 215.28	0.841	0.002^∗∗^
IP-10	7694.73 ± 5035.07	6382.49 ± 4539.37	<0.001^∗∗^	<0.001^∗∗^	7387.52 ± 4874.71	7335.05 ± 6475.22	<0.001^∗∗^	<0.001^∗∗^	367.60 ± 151.43	0.672	0.962
CXCL11 (I-TAC)	4116.99 ± 2659.76	1424.44 ± 1184.22	<0.001^∗∗^	<0.001^∗∗^	3725.09 ± 3105.49	1214.58 ± 1088.54	<0.001^∗∗^	<0.001^∗∗^	254.50 ± 531.58	0.199	0.326
IL-8	1911.43 ± 4409.65	1226.95 ± 2478.91	<0.001^∗∗^	0.004^∗∗^	2407.39 ± 5175.86	1793.00 ± 2336.76	<0.001^∗∗^	0.002^∗∗^	958.70 ± 2112.73	0.616	0.098
IL-10	45.88 ± 61.41	11.16 ± 11.80	<0.001^∗∗^	<0.001^∗∗^	60.11 ± 81.01	7.58 ± 5.07	<0.001^∗∗^	<0.001^∗∗^	1.50 ± 0.61	0.240	0.280
CCL2 (MCP-1)	9768.83 ± 9708.82	5807.54 ± 3414.33	<0.001^∗∗^	<0.001^∗∗^	8611.84 ± 6343.42	8009.00 ± 10517.43	<0.001^∗∗^	<0.001^∗∗^	2427.95 ± 845.28	0.809	0.339
IFN-*γ*	251.03 ± 917.31	15.05 ± 36.22	<0.001^∗∗^	<0.001^∗∗^	93.39 ± 152.62	7.79 ± 15.62	<0.001^∗∗^	0.054	0.35 ± 1.57	0.226	0.136
IL-18	205.80 ± 221.93	198.98 ± 211.30	0.005^∗∗^	0.047^∗^	286.20 ± 397.11	141.05 ± 115.95	0.029^∗^	0.325	105.45 ± 55.26	0.834	0.364
MIP-1*α*	244.68 ± 398.14	253.67 ± 431.30	0.006^∗∗^	0.003^∗∗^	223.41 ± 262.85	210.26 ± 157.82	0.156	0.002^∗∗^	127.20 ± 142.06	0.392	0.633
RANTES	11621.99 ± 4007.26	16017.28 ± 3794.66	<0.001^∗∗^	0.214	11632.41 ± 4207.56	17477.47 ± 3340.15	<0.001^∗∗^	0.933	17165.85 ± 2359.73	0.819	0.200
MIP-1*β*	196.97 ± 180.47	145.11 ± 188.44	0.237	0.511	165.84 ± 128.60	128.05 ± 100.70	0.845	0.491	146.85 ± 97.90	0.214	0.677
CCL20 (MIP-3*α*)	46.79 ± 198.24	21.38 ± 63.88	0.318	0.517	19.66 ± 27.61	15.84 ± 18.98	0.976	0.942	56.05 ± 178.53	0.212	0.401

Mean ± SD of cytokines were presented and performed analysis using Mann–Whitney *U* test or *t*-test between two cohorts. Statistical significance is indicated by asterisks: ^∗∗^*P* < 0.01 or ^∗^*P* < 0.05.

**Table 4:** Correlation between clinical routine indexes and cytokines.

	*R*	*P*
Primary infection		
UREA with		
RANTES	−0.131	0.049
CCL17	−0.196	0.003
CXCL5	−0.147	0.027
IL-8	−0.149	0.025
IL-10	0.132	0.048
CCL2	−0.151	0.023
Secondary infection		
PLT with		
CCL17	−0.314	0.012
LDH with		
MIP-1*α*	0.493	0.001
CXCL5	0.388	0.015
All infected patients		
UREA with		
RANTES	−0.126	0.033
CCL11	−0.137	0.020
CCL17	−0.167	0.005
CXCL5	−0.143	0.015
IL-8	−0.128	0.030
IL-10	−0.127	0.031
CCL2	−0.146	0.013
AST with		
CCL17	0.125	0.031
LDH with		
MIP-1*α*	0.186	0.006

Correlations between clinical routine indexes and cytokines were analyzed based on the Spearman rank correlation coefficient and *P* value. Only those with a *P* value <0.05 are presented in the table.
